# Rice Phospholipase A Superfamily: Organization, Phylogenetic and Expression Analysis during Abiotic Stresses and Development

**DOI:** 10.1371/journal.pone.0030947

**Published:** 2012-02-17

**Authors:** Amarjeet Singh, Vinay Baranwal, Alka Shankar, Poonam Kanwar, Rajeev Ranjan, Sandeep Yadav, Amita Pandey, Sanjay Kapoor, Girdhar K. Pandey

**Affiliations:** Department of Plant Molecular Biology, University of Delhi South Campus, New Delhi, India; Consejo Superior de Investigaciones Cientificas, Spain

## Abstract

**Background:**

Phospholipase A (PLA) is an important group of enzymes responsible for phospholipid hydrolysis in lipid signaling. PLAs have been implicated in abiotic stress signaling and developmental events in various plants species. Genome-wide analysis of PLA superfamily has been carried out in dicot plant *Arabidopsis*. A comprehensive genome-wide analysis of PLAs has not been presented yet in crop plant rice.

**Methodology/Principal Findings:**

A comprehensive bioinformatics analysis identified a total of 31 PLA encoding genes in the rice genome, which are divided into three classes; phospholipase A_1_ (PLA_1_), patatin like phospholipases (pPLA) and low molecular weight secretory phospholipase A_2_ (sPLA_2_) based on their sequences and phylogeny. A subset of 10 rice PLAs exhibited chromosomal duplication, emphasizing the role of duplication in the expansion of this gene family in rice. Microarray expression profiling revealed a number of PLA members expressing differentially and significantly under abiotic stresses and reproductive development. Comparative expression analysis with *Arabidopsis* PLAs revealed a high degree of functional conservation between the orthologs in two plant species, which also indicated the vital role of PLAs in stress signaling and plant development across different plant species. Moreover, sub-cellular localization of a few candidates suggests their differential localization and functional role in the lipid signaling.

**Conclusion/Significance:**

The comprehensive analysis and expression profiling would provide a critical platform for the functional characterization of the candidate PLA genes in crop plants.

## Introduction

Plants often encounter abiotic stresses such as high salinity, dehydration and low temperature during their life span. These abiotic stresses impose adverse growth conditions, which affect the plant development, longevity and productivity. The adaptive mechanism involves the activation of numerous signal transduction pathways, which lead to various molecular, cellular and physiological changes [1]–[4]. Recent findings propose an integral place for lipid signaling in complex regulatory network in response to abiotic stresses in plants [5]–[8]. Various environmental cues have been identified to trigger the hydrolysis of membrane phospholipids, which results in the generation of different classes of lipid and lipid-derived signal messengers such as phosphatidic acid (PA), diacylglycerol (DAG), DAG-pyrophosphate (DGPP), lysophospholipids, free fatty acids (FFAs), phosphoinositides and inositol polyphosphates [9]–[11]. Phospholipases are of utmost importance in lipid signaling as they are the essential enzymes for the catalysis of the initial step of phospholipids hydrolysis. Phospholipases have been broadly categorized as phospholipase A (PLA), phospholipase C (PLC) and phospholipase D (PLD) based on the action of respective enzyme at different sites on a glycerophospholipid molecule [12]. PLAs form an important group of lipid hydrolysing enzymes in plants. Based on their catalytic activity at the specific positions on a membrane glycerophospholipid, PLA superfamily has been divided into PLA_1_ and PLA_2_ subtypes. PLA_1_ catalyses the hydrolysis at *sn*-1 position of a phospholipid whereas PLA_2_ acts on *sn*-2 position. Products generated in the reaction such as FFA and lysophospholipids such as lysophosphatidylcholine (LPC) and lysophosphatidylethanolamine (LPE) are biologically active compounds, which are involved in several cellular signaling pathways [13]. PLA_1_ specifically hydrolyses phosphatidylcholine (PC) and PA-preferring PLA_1_ acts on PA. Depending on the particular sequences at N-terminal and similarity in the catalytic region, PC-hydrolysing PLA_1_s have been divided into groups I, II and III; and members of respective groups are predicted to be localized to chloroplast, cytosol and mitochondria, respectively [13]–[14]. Plant PLA_1_ members are characterised by the presence of a highly conserved GXSXG motif, a triad of three amino acids; Ser, Asp and His in the catalytic centre and a molecular weight of 45–50 kDa [15]–[17]. Sequence analysis also suggests three major groups of PLA_2_; calcium-dependent cytosolic phospholipase A_2_ (cPLA_2_), patatin like phospholipase A, which are homologous to animal calcium-independent phospholipase A_2_ (iPLA_2_) and low molecular weight secreted phospholipase A_2_ (sPLA_2_) [18]–[21]. However, cPLA_2_s are yet to be identified in plants [13]. Plant sPLA_2_s are characterised by the molecular weight of 13–18 kDa, PA2c domain, which comprises of a highly conserved Ca^2+^ binding loop (YGKYCGxxxxGC) and a catalytic site motif (DACCxxHDxC) with enzymatically active His/Asp residues [18], [22]–[24]. pPLAs are a group of vacuolar nonspecific lipid acyl hydrolases in solanaceae plants, possessing a combined PLA_1_ and PLA_2_ activity [13], [17], [25]–[26]. Based on the sequence analysis, plant pPLAs have been majorly categorised into three groups (I, II and III) [27] and recognized by the presence of a catalytic centre containing the esterase box (GTSTG) and the anion binding (DGGGXRG) motif. *Arabidopsis* genome exploration have resulted in the finding of twelve PC-hydrolysing PLA_1_ [14], one PA- preferring PLA_1_ [28], four sPLA_2_ [29]–[30] and ten pPLA members [20]. Moreover, two sPLA_2_ genes have also been reported in the rice genome [22].

In plants, different PLA members have been characterized and reported to be involved in seed development [31], root development [32], wounding and pathogen attack [33], hyperosmotic stress [1], cold and high salinity [34].

As evident by the survey of phospholipases in plants, most of the work has been carried out on dicot model plant *Arabidopsis* and some other species, but knowledge related to phospholipases A is minuscule in crop plant rice. Several individual reports proposed the involvement of these enzymes in numerous signaling networks and regulation of cellular processes such as stress signaling and tolerance, and development in dicot plant, especially *Arabidopsis*. It is quite obvious that these enzymes might also be involved in related functions in crop plant rice. Moreover, there is no report, which undertakes the investigation of expression of the PLA superfamily at the genome level. The rationale of a possible connection between the expression analysis and the functional role of PLAs *in planta* prompted us to undertake a comprehensive study towards the identification and transcript profiling of PLA superfamily in rice.

In the current study, we have identified the complement of PLA coding genes in the rice genome, which are found to be distributed into three main classes; PLA_1_, pPLA and sPLA_2_ based on the domain structures, conserved motifs and phylogenetic analysis. A detail expression analysis has been carried out under different abiotic stresses and during various stages of development. Subsequently, investigation has been done for the segmental and tandem duplication events and the sub-cellular localization for a few members.

## Results

### Identification and organization of PLAs in the rice genome

All the approaches employed for the mining of PLA encoding genes in the rice genome fetched a total of 31 genes. Domain and motif analysis using simple modular architecture research tool (SMART) (http://smart.embl-heidelberg.de/), InterPro (http://www.ebi.ac.uk/Tools/InterProScan) and Pfam (http://www.pfam.sanger.ac.uk/) tools could classify this set of genes into three major classes namely; PLA_1_, pPLAs and low molecular weight sPLA_2_. All the three PLA classes are represented by twelve, sixteen and three members, respectively in the rice genome (Table 1). Further, these PLA classes are divided into subgroups based on their protein sequences and domain structures. PLA_1_ class is sub-divided into PC-hydrolysing PLA_1_ and PA- preferring PLA_1_. PC-PLA_1_, based on the specific N-terminal sequences could be divided into subgroups I and II with eight and three members, respectively. We could not identify the group III member of this class in the rice genome. Similarly, pPLAs are divided into three subgroups I, II and III with one, nine and six members, respectively. We have identified three members belonging to sPLA_2_ group; two of these have been reported earlier [22]. Intron-exon analysis, carried out to study the gene structure revealed that the members of subgroup I of PC-PLA_1_ are intronless whereas subgroup II members contain 1–2 introns. Unlike *Arabidopsis* pPLAs, rice pPLAs revealed no set pattern of the gene structure for the members of respective subgroups [27]. Expression evidence could be obtained for approximately 50% of the genes in terms of full length cDNA and for 100% of the genes in terms of total ESTs (Table 1) and microarray based studies.

**Table 1:** Features of PLAs in the rice genome.

S.No.	TIGR Locus ID	Gene Name	FL cDNA	Total ESTs	Protein length(aa)	Intron	Putative localization
**PLA_1_**							
1	LOC_Os11g19340.1	*OsPLA_1_-Iα1*	AK119915	1	461	0	Chloroplast
2	LOC_Os01g67430.1	*OsPLA_1_-Iα2*	n/a	2	436	0	Chloroplast
3	LOC_Os11g19290.1	*OsPLA_1_-Iα3*	n/a	0	458	0	Chloroplast
4	LOC_Os01g67450.1	*OsPLA_1_-Iα4*	n/a	0	428	0	Chloroplast
5	LOC_Os02g43700.1	*OsPLA_1_-Iβ1*	AK106154	176	545	0	Chloroplast
6	LOC_Os08g04800.1	*OsPLA_1_-Iβ2*	n/a	2	482	0	Chloroplast
7	LOC_Os10g41270.1	*OsPLA_1_-Iβ3*	n/a	0	535	0	Chloroplast
8	LOC_Os05g32380.1	*OsPLA_1_-Iγ1*	AK289257	3	578	0	Chloroplast
9	LOC_Os01g46250.1	*OsPLA_1_-IIγ*	n/a	1	419	2	Cytosol
10	LOC_Os01g46290.1	*OsPLA_1_-IIβ*	AK102737	17	421	1	Cytosol
11	LOC_Os01g51360.1	*OsPLA_1_-IIδ*	AK069577	94	466	1	Cytosol
12	LOC_Os08g01920.1	*OsPA-PLA_1_*	AK099475	14	938	20	Nucleus
**pPLA**							
1	LOC_Os07g33670.1	*OspPLA-I*	AK243066	9	1227	1	Cytosol
2	LOC_Os09g28770.1	*OspPLA-IIα*	n/a	2	406	6	Cytosol
3	LOC_Os11g40009.1	*OspPLA-IIβ*	AK067827	17	246	2	Cytosol
4	LOC_Os08g37210.1	*OspPLA-IIγ*	CT828114	9	432	3	Chloroplast
5	LOC_Os08g37180.1	*OspPLA-IIδ*	AK101432	4	431	2	Chloroplast
6	LOC_Os11g39990.1	*OspPLA-IIε*	AK058531	20	418	4	Cytosol
7	LOC_Os08g28880.1	*OspPLA-IIζ*	n/a	1	443	3	Chloroplast
8	LOC_Os01g67310.1	*OspPLA-IIθ*	AK106563	49	412	5	Cytosol
9	LOC_Os03g27610.1	*OspPLA-IIϕ*	AK069809	15	433	3	Chloroplast
10	LOC_Os12g36530.1	*OspPLA-IIκ*	n/a	0	478	3	Cytosol
11	LOC_Os03g14950.1	*OspPLA-IIIα*	n/a	9	470	1	Nucleus
12	LOC_Os03g57080.1	*OspPLA-IIIβ*	n/a	1	463	2	Cytosol
13	LOC_Os07g05110.1	*OspPLA-IIIγ*	n/a	7	427	3	Cytosol
14	LOC_Os06g46350.1	*OspPLA-IIIδ*	n/a	4	405	2	Chloroplast
15	LOC_Os03g43880.1	*OspPLA-IIIε*	n/a	3	442	0	Others
16	LOC_Os12g41720.1	*OspPLA-IIIζ*	n/a	3	480	2	Others
**sPLA_2_s**							
1	LOC_Os03g50030.1	*OssPLA_2_α*	AK105828	29	164	3	ER
2	LOC_Os11g34440.1	*OssPLA_2_β*	CT835705	1	165	3	Cytosol
3	LOC_Os02g58500.1	*OssPLA_2_γ*	AK108410	5	139	2	Cytosol

### Phylogenetic and sequence analysis

**Figure 1:**
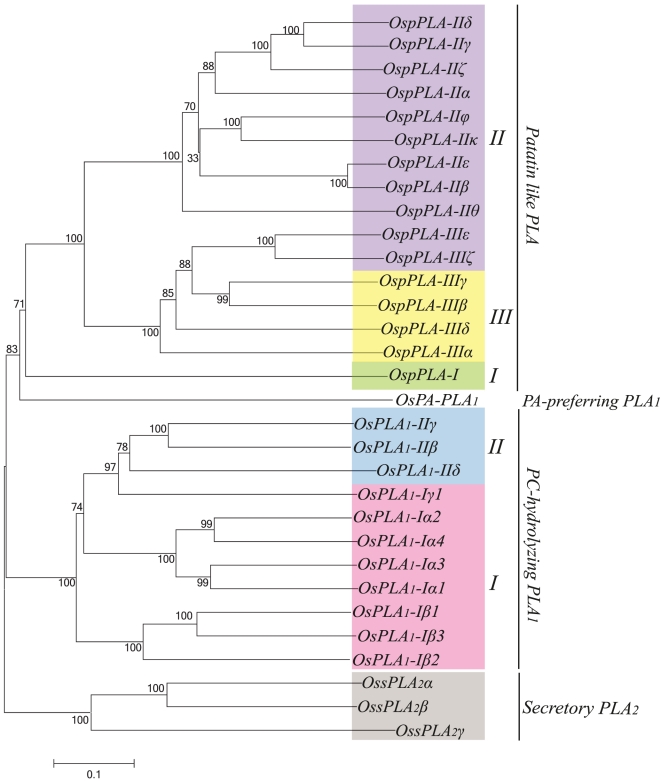
Phylogenetic relationship among various classes of PLA superfamily in rice. An un-rooted neighbor-joining tree was made from the protein sequences of all the PLA classes of rice. Multiple sequence alignment was done with ClustalX 2.0.8 and corresponding tree was generated in MEGA5. All the three PLA classes have been divided into subgroups (clades) indicated by different colors. Bootstrap value, out of 1000 replicates is indicated at each node. Scale bar represents 0.1 amino acid substitutions per site.

**Figure 2:**
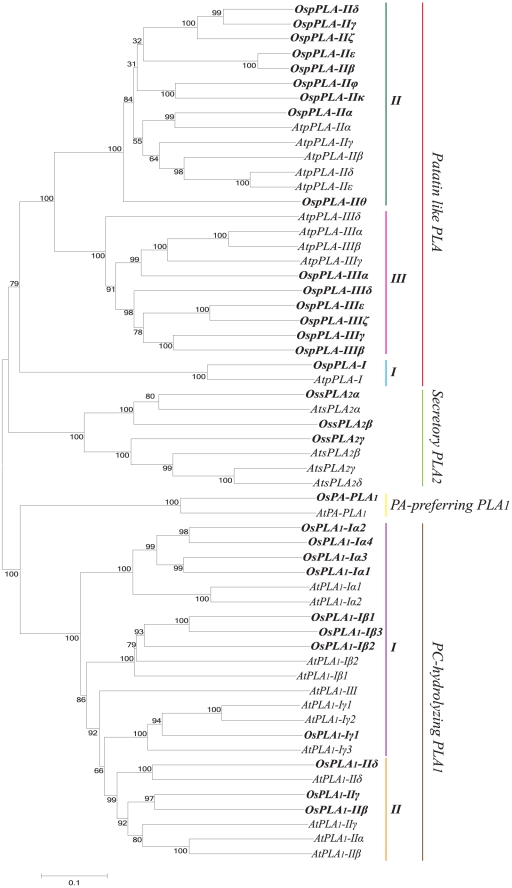
Phylogenetic relationship between rice and *Arabidopsis* PLA superfamily. An un-rooted neighbor-joining tree was made from the total protein sequences of rice and *Arabidopsis* PLAs (PLA_1_, pPLA and sPLA_2_). Multiple sequence alignment was done using clustalX 2.0.8 and tree was generated using MEGA5. PLAs from rice and *Arabidopsis* are grouped, based on the bootstrap support value ≥50%. Scale bar represents 0.1 amino acid substitutions per site.

**Figure 3:**
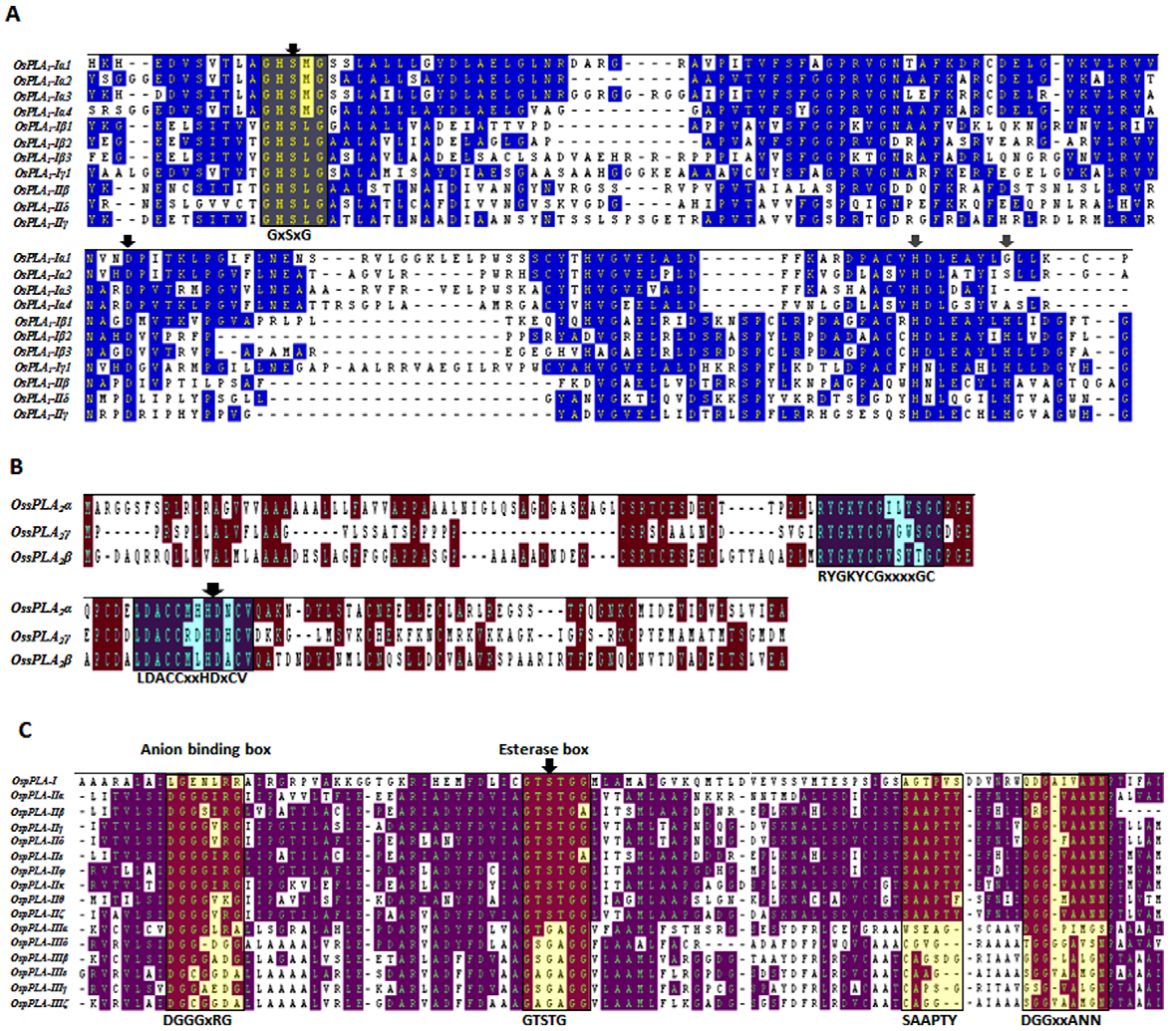
Multiple sequence alignment of (A) OsPLA_1_ (B) OssPLA_2_ and (C) OspPLA showing the consensus and conserved motifs. Protein sequences were aligned for each PLA class, separately applying clustalW tool of Megalign-DNA STAR. The consensus and conserved sequences have been shown in shadow boxes. Catalytically important residues have been indicated by the black arrowheads. Putative His residues of catalytic centre in PLA_1_ (A) have been indicated by gray arrowheads.

Phylogenetic analysis was performed for all the rice PLAs using protein sequences to find out the evolutionary relatedness among various groups. Based on the statistical analysis of ≥50% bootstrap support, all the three PLA classes are divided into different clades representing subgroups (Figure 1). Members of the same subgroup in a PLA class seemed to have high degree of evolutionary relatedness and some divergence from the members of the other subgroups. Investigation for the evolution of this set of genes between two plant species, rice and *Arabidopsis* revealed that the members of respective subgroups from two different plant species were falling into the same clades with high degree of relatedness (Figure 2). This pattern suggests the common ancestry of PLAs in the two diverse plant species, and it is used for the classification and nomenclature of the genes in rice. Multiple sequence alignment analysis of different PLA classes has confirmed the presence of highly conserved regulatory and catalytically important motifs. All PLA_1_ members had a highly conserved GXSXG motif and a catalytic centre of Ser, Asp and His residues. Consistent with the previous findings, pPLA members were found to harbour the characteristic esterase box GTSTG and the anion binding DGGGXRG motif. However, in group III, serine of the catalytic centre (esterase box) is replaced with glycine. Similarly, sPLA_2_s harboured a defined PA2c domain with a highly conserved Ca^2+^ binding loop denoted by YGKYCGxxxxGC motif and the catalytic site LDACCxxHDxCV with enzymatically active His/Asp residues (Figure 3). Analysis of the sequences for the putative subcellular localization using *in-silico* tools such as Target P 1.1 [35] and WoLF PSORT (http://wolfpsort.org/) [36], indicated the localization of different PLAs to various organelles (Table 1). In accordance with the previous studies [14], members of group I of PC-PLA_1_ have been localized to chloroplast and group II members localized to cytosol.

### Chromosomal localization and gene duplication

**Figure 4:**
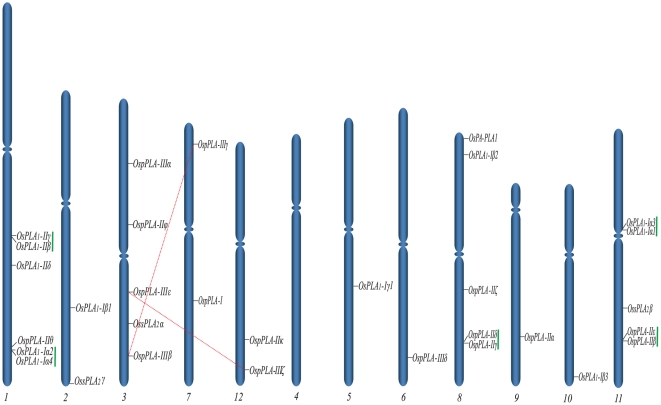
Chromosomal localization of PLA genes on 12 chromosomes of rice. Genes from different PLA classes have been mapped by their position on 12 chromosomes. Dashed red lines join the genes, lying on duplicated segments of the genome. Tandemly duplicated genes are joined with vertical green lines. Chromosomes are grouped randomly to show the duplication with clarity. No PLA gene was localized to chromosome 4. Respective chromosome numbers are mentioned at the bottom.

Rice PLAs were mapped to rice genome annotation project (RGAP) (http://rice.plantbiology.msu.edu/) pseudomolecules (version 6.1; chromosome 1–12), depending on the coordinates of RGAP loci (http://rice.plantbiology.msu.edu/pseudomolecules/info.shtml). PLA encoding genes were found to be variably distributed on the rice chromosomes, where the chromosome 4 being exception and none of the PLA gene was located on it. A maximum of six genes were located on the largest rice chromosome (chromosome 1); followed by the chromosomes 3, 8 and 11, five PLA genes are localized to each. A single PLA gene was found to be localized on chromosomes 5, 6, 9 and 10 each (Figure 4). A pair of genes belonging to pPLAs (*OspPLAIIIζ:OspPLAIIIε*) was found to be segmentally duplicated. On the other hand, there were ten genes (five pairs), which were marked as tandemly duplicated, based on the criterion of separation by less than five intervening genes. Among the tandemly duplicated genes, three pairs belonged to PLA_1_ class and two pairs to pPLAs (Table S1). None of the sPLA_2_ members were found to be duplicated.

### Expression profile of rice PLAs under abiotic stress conditions

**Figure 5:**
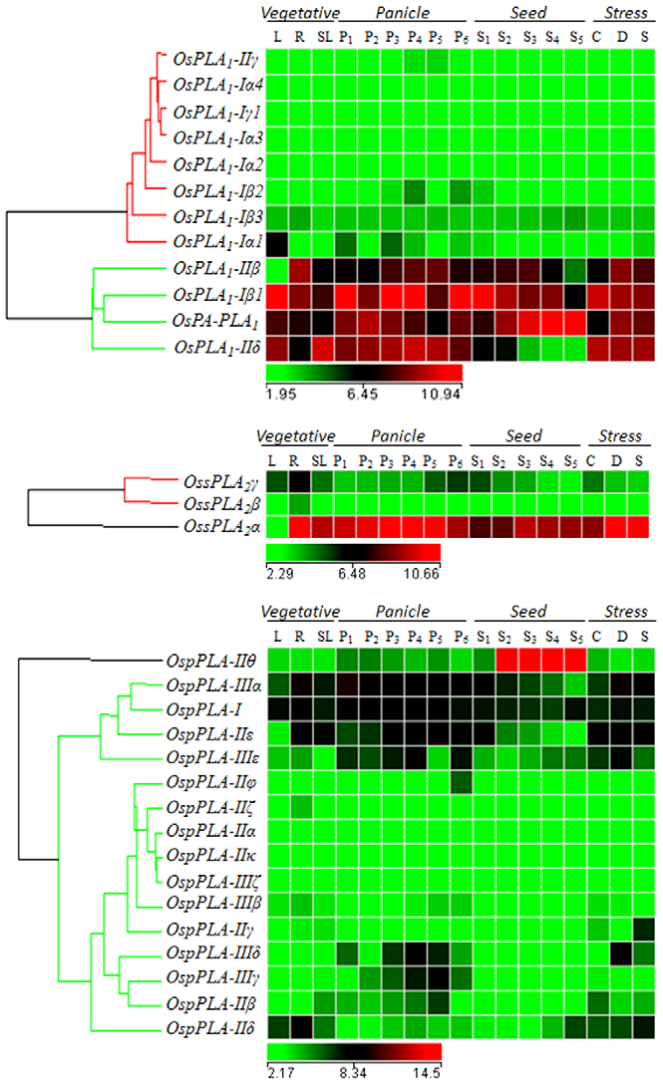
Expression profiles of rice PLA superfamily. Different PLA class expression has been represented by a separate heat map. Reproductive development comprising six stages of panicle [P1 (0–3 cm), P2 (3–5 cm), P3 (5–10 cm), P4 (10–15 cm), P5 (15–22 cm), and P6 (22–30 cm)] and five stages of seed [S1 (0–2 DAP), S2 (3–4 DAP), S3 (4–10 DAP), S4 (11–20 DAP) and S5 (21–29 DAP)]. Clustering of the expression profile was done with log transformed average values taking mature leaf as base line. Three experimental stress conditions are denoted as C: Cold Stress, D: Drought Stress, S: Salt Stress and SL: control, seven day old unstressed seedling. A gene is considered differentially expressed during reproductive development if up- or down-regulated at least two-fold, with respect to the three vegetative controls (mature leaf, root and 7- day old seedling) and with respect to the seven day old unstressed seedling in case of abiotic stress. The color scale at the bottom of each heat map is given in log_2_ intensity value.

Expression profile of the PLA encoding genes was generated using microarray data obtained from Affymetrix rice genome arrays for seven day old rice seedling treated for three abiotic stress conditions (salt, cold and drought). A cut off value of fold change ≥2 (either up- or down-regulated) in comparison to untreated seven day old seedling, was set to define a gene to be differentially regulated. Based on this criterion, a total of nine genes were found to be significantly and differentially expressing under three abiotic stresses (Figure 5, Table S2). This subset of genes included a member from all the three PLA classes (three-PLA_1_, five-pPLA and one-sPLA_2_). Interestingly, all the differentially expressed genes were found to be up-regulated under a specific or multiple abiotic stresses. Two genes, *OsPLA_1_-Iβ1* and *OspPLA-IIIε* were commonly up-regulated in all three stress conditions. Four genes, *OsPLA_1_-IIβ*, *OsPA-PLA_1_*, *OspPLA-IIIδ* and *OspPLA-IIγ* were commonly up-regulated in salt and drought stresses together, whereas none of the genes were commonly expressed under cold and salt or cold and drought stresses together. Observation for the genes, expressing exclusively under any one of the abiotic stresses, revealed two (*OspPLA-IIIα and OssPLA_2_α*) and a single (*OspPLA-IIδ*) genes being up-regulated in drought and salt stress, respectively. None of the PLA genes were found to be exclusively expressed under cold stress (Table S2).

### Expression profile of rice PLAs during development

Expression analysis for the development was performed by studying 11 stages including, six panicle (P1–P6) and five seed (S1–S5) reproductive stages, compared with three combined vegetative developmental stages namely leaf, root and seedling as described in Arora et al., 2007 [37]. A total of 19 genes were differentially expressed with a fold change value ≥2 (either up- or down-regulated), during various reproductive developmental stages (Figure 5, Table S3). The group of differentially expressing genes included six PLA_1_s, ten pPLAs and three sPLA_2_s. Out of these, fifteen and four genes were up-and down-regulated, respectively, during panicle and seed developmental stages. A subset of eight and three genes were commonly up-regulated and down-regulated, respectively, in both panicle and seed developmental stages. The transcript levels of six genes were escalated and for a single gene it was declined in the panicle stages exclusively. On the other hand, the transcript levels were declined for three genes in seed stages exclusively, whereas none of the genes showed increased transcript level. Interestingly, these three down-regulated genes (*OsPLA_1_-IIδ*, *OsPLA_1_-Iα1* and *OspPLA-IIβ*) in seed stages, belonged to the subset of 6 genes, which were exclusively up-regulated in panicle stages (Table S3). Since stress tolerance and reproductive development are closely related phenomenon of plant life cycle, we have made an attempt to investigate such relationship in the signaling pathways where PLAs are involved. Therefore, we have analysed the expression profile of the PLA genes during reproductive development and under various abiotic stresses. Surprisingly, out of nine genes expressed differentially under abiotic stresses, eight genes were also involved in the developmental stages with significant expression profile. Out of this subset of eight genes, six were found to be commonly up-regulated under abiotic stresses and both flower and seed development. A single gene *OspPLA-IIδ*, which was up-regulated in abiotic stresses, was found to be down-regulated in developmental stages (Table S2, S3).

### Comparative expression analysis of rice and Arabidopsis PLAs

To correlate the evolutionary and structural conservation of this superfamily with the functional conservation in different plant species, a parallel comparative expression analysis was carried out for rice and *Arabidopsis* PLA superfamily under three abiotic stresses (salt, cold and drought). The analysis showed that a high proportion of *AtPLA* genes were inducible under similar abiotic stress conditions as *OsPLAs*. Significant changes in the transcript level (either up- or down-regulation) were observed for nine out of 13 *AtPLA_1_* members, under one or multiple abiotic stresses (especially salt and drought). Eight out of ten *AtpPLAs* and a single secretory PLA_2_, *AtsPLA_2_α* were found to be differentially expressed, under one or more abiotic stresses (Figure S1). Similarly, transcript analysis in various *Arabidopsis* tissues such as seedling, leaf and root (vegetative), seed and floral organs (reproductive) revealed that the transcript levels were differentially altered for seven PLA_1_s and seven pPLAs, when compared the reproductive tissues with the vegetative tissues (Figure S2). Here, we could find a functional conservation between a number of PLA orthologs of rice and *Arabidopsis*. Genes such as *AtPLA_1_-Iβ1*, *AtPLA_1_-IIβ*, *AtPLA_1_-IIδ*, *AtpPLA-IIIα* and *AtpPLA-IIIβ* were specifically marked for having similar expression pattern, in similar tissues both in monocot (rice) and dicot (*Arabidopsis*) plant species.

### Expression profile of duplicated rice PLA genes

**Figure 6:**
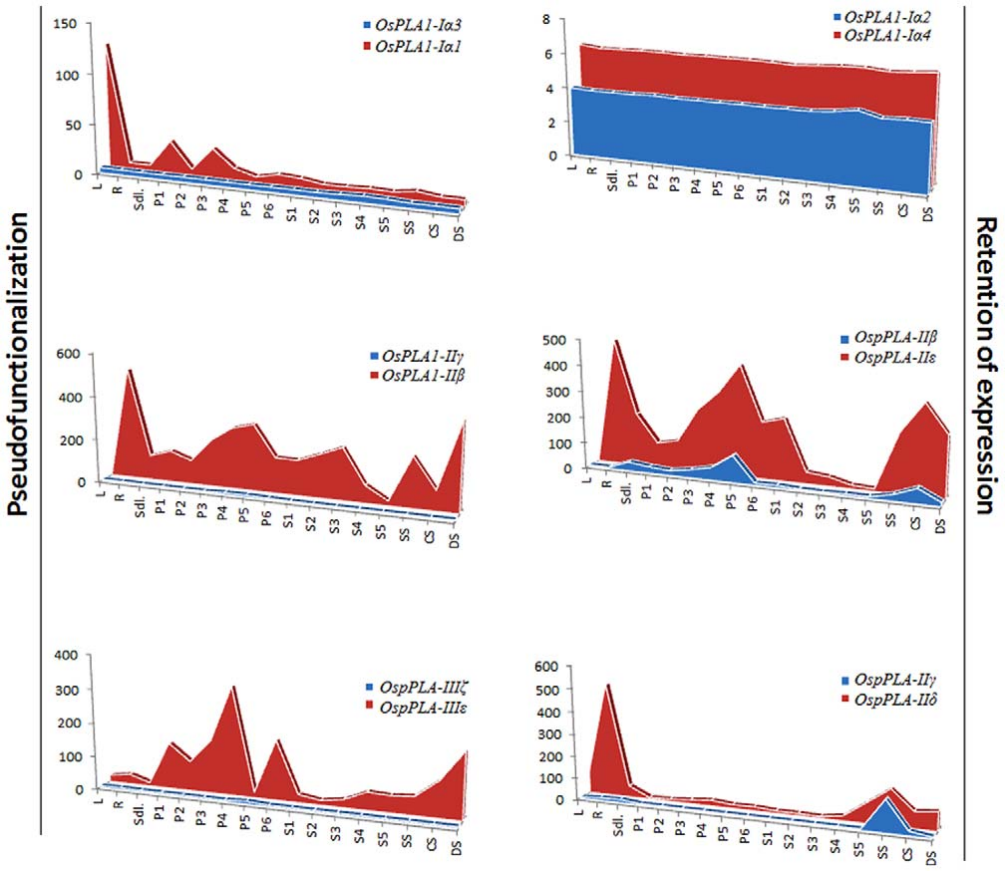
Expression pattern of duplicated PLA genes. The expression values of segmentally and tandemly duplicated genes obtained from microarray data were compared in leaf (L), root (R) and seven day old seedling (SDL) tissue, in various stages of panicle development (P1–P6), seed development (S1–S5) and under cold stress (CS), dehydration stress (DS) and salt stress (SS). Each area graph represents compilation of the mean normalized signal intensity values from 17 stages of development/stress conditions. Gene pairs have been grouped into retention of expression and pseudo-functionalization based on their respective profile.

An expression profile for duplicated *OsPLA* genes was also generated from microarray data for abiotic stresses and development. The average signal values for all the samples (abiotic stresses as well as developmental stages) are presented as an area-diagram (Figure 6). A single pair of segmentally duplicated genes, *OspPLA-IIIζ:OspPLA-IIIε* and two pairs of tandemly duplicated *OsPLAs* (*OsPLA_1_-Iα1*:*OsPLA_1_-Iα3* and *OsPLA_1_-IIγ*:*OsPLA_1_-IIβ*) exhibited pseudo-functionalization, as one of the duplicated partner had negligible expression. In case of other three tandemly duplicated PLA gene pairs, both the genes in a pair followed the similar expression pattern, throughout the spectrum of conditions and stages, hence, showed retention of expression (Figure 6). However, the magnitude of the expression varied between the duplicated partners.

### Q-PCR validation of microarray expression under abiotic stress conditions

**Figure 7:**
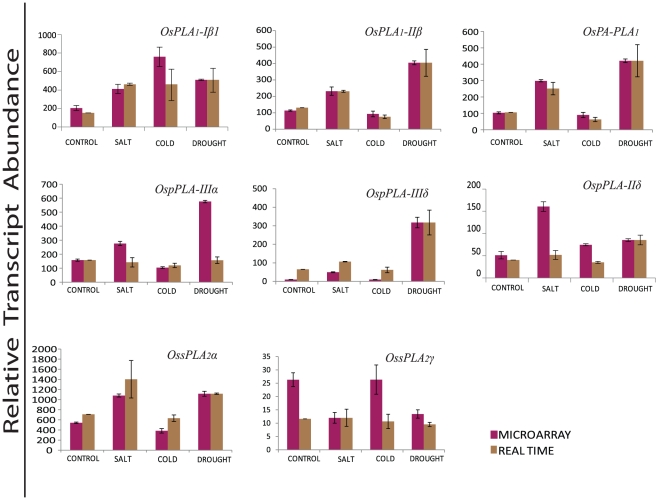
Validation of microarray expression profiles for selected PLA candidate genes by quantitative RT-PCR. Real time PCR and microarray analysis was performed taking two and three biological replicates, respectively. Standard error bars have been shown for data obtained both from microarray and real time PCR. Y-axis represents raw expression values from microarray and normalized expression value from real time PCR and X-axis shows different experimental conditions; purple bars represent the expression from microarrays, while brown bars represent the real-time PCR values.

Among the rice PLA genes, which were found to express significantly and differentially under various abiotic stresses by microarray analysis, the expression pattern have been validated for eight genes, employing quantitative real time PCR. This subset of genes comprises the members from all the three PLA classes (three PLA_1_, three pPLA and two sPLA_2_). Out of these, seven candidate genes exhibited expected expression pattern and showed significant variation in the transcript level w.r.t control, when subjected to different abiotic stresses. However, the magnitude of expression varied slightly in most of the genes and samples tested. The expression profile of a single gene *OspPLA-IIIδ*, deviated from the anticipated expression pattern and showed up-regulation, in contrary to its down-regulation shown by microarray under the cold stress treatment (Figure 7).

### Subcellular localization of rice PLAs

**Figure 8:**
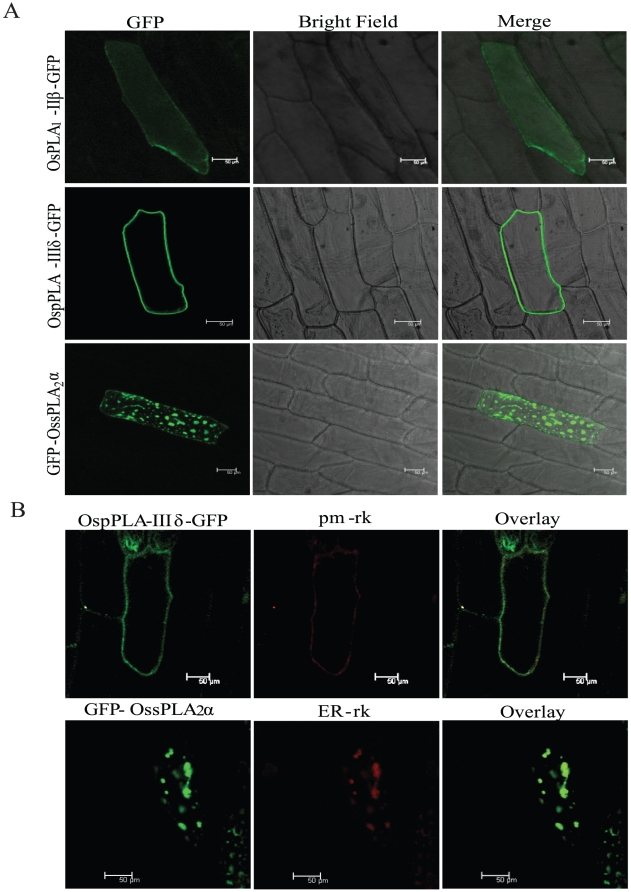
Sub-cellular localization of OsPLA proteins. (A). Onion peel epidermal cells showing expression of the OsPLA - green fluorescent (GFP) fusion protein driven by the *35S* promoter. Confocal images of fluorescence are shown for the expression of OsPLA_1_-IIβ-GFP fusion protein, showing its distribution throughout the cytoplasm (upper row), expression of OspPLA-IIIδ-GFP fusion protein showing their preferential accumulation in cell periphery (middle row) and of GFP-OssPLA-2α fusion in specific organelle in the cell (lower row). All the images were taken in 5 different sections in z direction and merges together. Scale bar = 50 µm. (B). Co-localization of PLA proteins with organelle markers. OspPLA-IIIδ-GFP showing GFP signal merges completely with plasma membrane marker (pm-rk) (upper panel) and GFP-OssPLA-2α was present as a large bright spot that co-localize with endoplasmic reticulum (ER) marker (ER-rk) (lower panel). GFP fusion to the OsPLA proteins are shown in green, mCherry organelle markers are shown in red and overlay of two mentioned protein in dark field view. All the images were taken in 5 different sections in z direction and merges together. Scale bar = 50 µm.

Onion peel epidermal cells were transformed transiently by particle bombardment method to determine the GFP fusion protein expression and subcellular localization, for three PLA members; OsPLA_1_-IIβ, OspPLA-IIIδ and OssPLA_2_α, representing all the three PLA classes. It was observed that OsPLA_1_-IIβ has been distributed throughout the cytoplasm as seen by GFP fluorescence (Figure 8A). OssPLA_2_α was observed as thick patches within perinuclear and cortile region of the cell, and assumed to be localized to ER. Fluorescence for OspPLA-IIIδ was observed in the periphery of the cell, and it seems to be localized at the plasma membrane (Figure 8A). For further confirmation, co-localization of GFP-OssPLA_2_α protein has been done with an endoplasmic marker ER-rk (CD3-959, ABRC) and OspPLA-IIIδ-GFP with plasma membrane marker pm-rk (CD3-1007, ABRC) [38]. It was observed that GFP signal of GFP-OssPLA_2_α overlaps with ER-rk fluorescence signal and that of OspPLA-IIIδ-GFP with pm-rk tracker (as shown in pseudo colors green and red, respectively) (Figure 8B). Hence, confirmed the localization of OssPLA2α and OspPLA-IIIδ to ER and plasma membrane, respectively.

## Discussion

### Organization of Phospholipase A in rice

Genome wide exploration with various approaches has culminated in the identification of 31 PLA encoding genes in the rice genome. Protein sequence, domain structure and phylogenetic analysis have facilitated the classification of this superfamily into three major classes; PLA_1_, pPLA and low molecular weight sPLA_2_, each represented by 12, 16 and 3 members, respectively. Earlier, the group of genes encoding these enzymes have been reported in the *Arabidopsis* genome where, 12 PLA_1_ [14], 10 pPLAs [20] and 4 members of sPLA_2_ group [29]–[30] were identified. We could identify a single PA-PLA_1_ encoding gene in the rice genome (included in 12 PLA_1_), an ortholog of which has been identified in *Arabidopsis* [28] and bovine testis [39]. Domain analysis by SMART, InterPro and Pfam could mark the presence of “lipase3” in PLA_1_ (PC-PLA_1_) members, “DDHD” domain in PA-PLA_1_, “patatin” in pPLAs and “PA2c” domain in all the sPLA_2_s, the characteristic features of various plant PLA classes [13]. Also, the highly conserved signature motifs for different groups of PLAs have been identified in rice (Figure 3), which were previously reported in *Arabidopsis* [14], [20], [23]. However, in group III of rice pPLAs, serine of the catalytic centre is replaced by glycine. This kind of replacement in the amino acid residue of pPLA group III has been observed previously [17]. The observation for the putative sub-cellular localization by *in-silico* tools indicated the variable distribution of OsPLAs to chloroplast, mitochondria, cytoplasm and other orgenelles (Table 1). More importantly, the members of OsPC-PLA_1_ class followed the localization pattern of *Arabidopsis* PC-PLA_1_s [14]. The entire group I members have been localized to chloroplast and that of group II localized to cytosol. Intron-exon or gene structure analysis revealed that PC-PLA_1_s are structurally conserved in different plant species as group I members of this class were found to be intronless (except *AtPLA_1_-Iγ1*), whereas group II members contained one to two introns both in rice (Table 1) and *Arabidopsis*. However, pPLAs differred in their intron-exon structure from their orthologs in *Arabidopsis* [27]. Chromosomal mapping of rice PLA genes showed their variable distribution on 11 rice chromosomes, excluding chromosome 4. Interestingly, there were five gene pairs in tandem duplication, localized on chromosome 1, 8 and 11 (two, one and two pairs, respectively) and a single pair *OspPLAIIIζ:OspPLAIIIε*, represented as duplicated segments between chromosome 3 and 12 (Figure 4). This observation suggests a role for the chromosomal duplication in the expansion and evolution of various PLA groups and the PLA superfamily in rice.

### PLAs are evolutionary conserved in rice and Arabidopsis

Phylogenetic analysis demarcated different groups and indicated a high degree of relatedness, among the members of various groups of different PLA classes in rice (Figure 1). Evolutionary studies in rice and *Arabidopsis* PLAs indicated a common ancestry and conserved evolution of this group of genes, in two different plant species from monocots and eudicots, as the members from different PLA groups from these different plant species falls in the same phylogenetic clades (Figure 2). This evolutionary information can be extrapolated to the functional conservation of various PLA genes in different plant species and hence could serve as a useful tool to assign putative function for the rice PLA members. Moreover, rice PLA genes such as *OsPLA_1_-Iβ1* has its *Arabidopsis* ortholog *AtPLA_1_-Iβ1* (*DEFECTIVE IN ANTHER DEHISCENCE1-DAD1*), which play role in jasmonic acid (JA) biosynthesis, pollen maturation, anther dehiscence, and flower opening in *Arabidopsis* [14]. Similarly, *OsPA-PLA_1_* orthologous gene *AtPA-PLA_1_* (also known as *SGR2*) reported to have a role in shoot gravitropism [28], [40] and in vesicular trafficking in eukaryotic cells [41]. *AtsPLA_2_-β* has been implicated in auxin signaling and found to be responsible for cell elongation by stimulating cell wall acidification [30]. pPLAs have also been implicated in various functions in plants. Overexpression of *pPLA-IIα* augmented the plant cell death and is also implicated in the tolerance against pathogen attacks in *Arabidopsis* [42]. The knockout study of *pPLA-IIγ* in *Arabidopsis* has revealed that this gene is required for the root response to phosphate deficiency [32]. Similarly, the overexpression of *pPLA-IIIδ*, lead to an STURDY mutant, which was marked by a stiff inflorescence stem, thick leaves, short siliques, large seeds, round flowers and delayed growth [43]. Since rice PLAs share high sequence homology with *Arabidopsis* PLAs, they can also be speculated to be involved in various functions portrayed by *AtPLAs*. Apart from all the mentioned physiological and cellular processes, abiotic stress signaling and tolerance are important aspects, which affect the plant growth and development, the lifespan and ultimately the yield in case of crop plants.

### Role of PLAs in abiotic stress signaling and plant development

An expression profile of a gene provides a clue about its functional relevance. This fact enticed us to generate a genome wide expression profile of PLAs under different abiotic stresses and developmental stages in rice. In our genome wide microarray expression analysis, a subset of PLA genes including different classes, showed significant and differential expression pattern under three abiotic stresses and during various stages of reproductive development (Table S2, S3). The expression profile of eight selected candidate genes was generated by quantitative real time PCR, and it showed that most of the PLA genes followed the microarray expression pattern, validating the transcript level of various rice PLA genes, with or without the abiotic stress trigger in rice seedlings. Moreover, a comparative transcript analysis for rice and *Arabidopsis* PLAs in similar stress conditions and developmental stages/tissues (Figure S1, S2), showed that this group of genes have similar expression profiles, which could be correlated in the orthologs in many cases in the two plant species. This also suggests that high conservation at the sequence level, same evolutionary path and common ancestry (shown by the phylogenetic analysis) of PLA superfamily in monocots and dicots, might have accounted for the functional conservation in this group of genes, across the plant species. This fact is indicative of involvement of various PLA members in different abiotic stress triggered signaling pathways and in various tissues and development of the plants. Previously, by northern blot analysis, the transcript level of a pPLA was shown to be increased, in the leaves of tropical legume *Vigna unguiculata* L. Walp (cowpea) in response to drought [44]. *PLA IIA* from *Arabidopsis* was found to be induced in response to abiotic stresses, including cold and high salinity [34] and PLA_2_ mediated lyso-phophatidic acid synthesis was shown to be induced rapidly in response to salt and other osmolytes in *Chlamydomonas* [1]. Similarly, three members of *Arabidopsis* patatin like phospholipases; *AtPAT IIA*, *AtPAT IVC* and *AtPAT IIIA* were up-regulated, when subjected to water deficit conditions, shown by RT-PCR analysis [45]. PLA members have also been implicated in various developmental events in plants. It has been reported that PLA_2_ mediate the auxin induced cell elongation in plants [46]–[47] and *AtsPLA_2_β* has been found to be involved in such process [30]. Similarly, a patatin like protein was detected in the developing seeds of cucumber [31]. Recently, in a knockout mutant study, three members of *Arabidopsis* patatin family; *AtPLA IVA*, *AtPLA IVB*, and *AtPLA IVC* were found to be associated with root development [32]. In our expression analysis, we could identify several PLA members with overlapping expression, under abiotic stresses and during stages of reproductive development. This type of overlapping expression has been reported earlier in various plant gene families [48]–[50] and it could be the result of some *cis*-regulatory elements such as ABRE, controlling both stress and development together [50]. Moreover, it has been well recognized that the plant developmental processes and abiotic stress signaling are interconnected phenomenon [49]–[51]. A programmed dehydration event is known to be triggered in plants during the later stages of seed maturation leading to seed dormancy [48], [51]–[52]. The phytohormone such as ABA, commonly mediates these events of plant development and abiotic stress [53]–[55]. Therefore, a correlation can be anticipated, where similar physiological event might be taking place in plants, involving concerted function of PLAs and ABA.

### Duplication has contributed to the expansion and functional diversification of PLAs in rice

We have generated the expression profiles for all the duplicated rice PLA genes, covering the spectrum of abiotic stresses and vegetative as well as reproductive development phases. Duplicated PLAs have exhibited varied expression pattern under abiotic stresses and during development, as duplicated partners differ in their expression. The variable expression might have resulted due to the absence of intense selection pressure and might have been needed for the diversification [56]–[59]. It has been documented previously that the segmentally duplicated genes display a great degree of functional divergence [57]. Consistent with this finding, out of five pairs of tandemly duplicated PLAs, two pairs exhibited pseudo-functionalization and three pairs exhibited retention of expression. Whereas, a single segmentally duplicated pair showed pseudo-functionalization, as one of the paired partner had almost negligible expression throughout the spectrum of abiotic stresses and the stages of development (Figure 6). All the three pairs of genes retaining their expression, had 63%–91.8% similarity at the amino acid level between the partners. This high level of homology might have accounted for the similarity in their expression pattern. Other three pairs, which showed pseudo-functionalization also had a high amino acid homology (51.2%–72%), indicating that these genes might have undergone tremendous diversification after duplication and one of the partners has completely lost its function during the evolution. These observations suggest that the chromosomal duplication has been one of the potent forces, which have driven the evolution of PLA superfamily in crop plant rice.

### Sub-cellular localization suggests functional role of PLAs in various cellular processes

Genome-wide expression analysis has yielded valuable information regarding the expression at transcript level. Expression of a protein in the cell can be studied by tagging the protein with fluorescent tag such as GFP/RFP/YFP/CFP etc. and then visualizing its localization at the sub-cellular level. In the present study, successful localization of the members from each class of rice PLAs in onion epidermal peel cells indicates that these PLAs encode functional proteins. Furthermore, sub-cellular localization of few OsPLA members has provided an insight towards their site of action and functional importance in various cellular processes. The localization of OssPLA_2_α to ER has been consistent with the earlier reports on *Arabidopsis* PLA_2_, suggesting its role in protein trafficking and pollen development [60]–[61]. Similarly, localization of PLA_1_ (OsPLA_1_-IIβ) to cytoplasm and patatin like phospholipase (OspPLA-IIIδ) to plasma membrane have been in accordance with the previous findings [13], [62] where, *Arabidopsis* pPLA-IIIβ protein was observed in plasma membrane and alteration in its expression modulated the cellulose content and cell elongation. The diverse sub-cellular localization of different groups of rice PLAs, suggests a significant role of OsPLAs in multiple cellular processes.

### Conclusions

In conclusion, this study, for the first time presents a comprehensive account of PLA encoding genes in rice, their genomic organization and phylogenetic relationship. Expression profiling of PLA genes has proposed their probable function in abiotic stress signaling and development. A comparative phylogenetic and expressional analysis of PLAs between eudicot *Arabidopsis* and monocot rice have identified the significant functional conservation in these two diverse plant species. Moreover, the differential sub-cellular localization of a few candidate PLAs also suggests the possible involvement of these candidates in diverse lipid signaling events in the plant cell. This study opens a field for detail molecular characterization and investigation to ascertain the *in-planta* role played by PLAs in crop plant rice.

## Materials and Methods

### Identification of PLAs in the rice genome

Putative PLA candidates were searched in the RGAP version 6.1 and in NCBI using different keywords, and by phospholipase A superfamily domain annotations in SUPERFAMILY database (http://supfam.cs.bris.ac.uk/SUPERFAMILY). Hidden Markov Model (HMM) profiles corresponding to different PLA classes were obtained by seed alignment at default parameters (E value – 1.0) from Pfam and used as query to search RGAP protein database.

*Arabidopsis* PLA members were searched and the sequences were downloaded from TAIR 10.0 (The *Arabidopsis* Information Resource) and by referring to the published literature [13], [27]. *Arabidopsis* sequences were used as query to perform the homology search both at protein and nucleotide level using BLASTP and BLASTN, respectively, in RGAP 6.1 and NCBI. Putative rice PLA sequences with >60% identity at nucleotide level and >90% identity at amino acid level were retained for further analysis. The results obtained from all these approaches (keyword, HMM and homology search) were merged together and unique entries (with unique locus ID) were identified to remove the redundancy of genes. Further confirmation was done by scanning the putative sequences through SMART, InterPro and Pfam search tools, for the presence of characteristic domains, and the signature motifs, which categorised them into different PLA classes.

### Protein sequence alignment and phylogenetic analysis

The protein sequences of all the non-redundant candidates were used for multiple sequence alignment using clustalX (version 2.0.8) and a maximum likelihood, un-rooted phylogenetic tree was constructed to infer the evolutionary history for all the rice PLA class. The tree was generated by neighbour-joining (NJ) algorithm with p-distance method and pairwise deletion of gaps, employing MEGA version 5 [63], using default parameters. Phylogenetic tree was also generated for rice and *Arabidopsis* PLAs together to find out the sequence conservation and functional homology in two different plant species. A bootstrap statistical analysis was performed with 1000 replicates to test the phylogeny.

### Sequence analysis

The amino acid sequences of all the PLA classes were aligned and analysed for the conserved regulatory and catalytically important motifs, employing MegAlign software 5.07^©^ [64] using ClustalW method. Also, the protein sequences were analysed for the putative subcellular localization by TargetP1.1. and WoLF PSORT tools.

### Gene nomenclature and attributes

Gene names have been assigned to the members of different PLA classes, based on their phylogenetic relationship with their *Arabidopsis* orthologs, protein sequence and domain structures. Similarly, different PLA classes could be divided into subgroups, with the convention followed in case of *Arabidopsis*. Various gene attributes such as locus ID, protein (aa) size, introns, and expression evidences in terms of total number of ESTs and full length cDNA were extracted from RGAP ver. 6.1 and knowledge based oryza molecular biological encyclopedia (KOME) (http://cdna01.dna.affrc.go.jp/cDNA) for all the PLAs.

### Chromosomal localization and gene duplication

Genes were mapped onto chromosomes by identifying their positions as given in RGAP pseudomolecules (version 6.1; chromosome 1–12) according to the coordinates of RGAP loci (http://rice.plantbiology.msu.edu/pseudomolecules/info.shtml). The RGAP segmental duplication database (http://rice.plantbiology.msu.edu/segmental_dup/500kb/segdup_500kb.shtml) was explored to find out the duplicated genes. Genes separated by five or fewer genes were considered as tandemly duplicated. The amino acid sequence homology between the products of tandemly duplicated genes was computed using MegAlign software.

### Plant growth and abiotic stress treatment

The tissues were harvested from field grown rice plants (*Oryza sativa* ssp. *Indica* var. IR64), at different stages of panicle and seed development [65]. In order to prevent from wounding, collected panicles were immediately frozen in liquid nitrogen. For stress treatment, sterilized IR64 rice seeds were grown in culture room conditions at 28±1°C with a daily photoperiodic cycle of 14 h light and 10 h dark. After seven days growth, different stress treatments were given to the seedlings. Salt treatment was given by transferring the seedlings into 200 mM NaCl solution, for cold treatment, seedlings were kept at 4±1°C on sterile water and for dehydration, seedlings were air dried on a Whatman sheet at 28±1°C for 3 h. Parallel control samples were prepared by keeping the seedlings on water for 3 h. Treated seedlings were immediately frozen in liquid nitrogen.

### Microarray experiments

Microarray experiments were carried out for three vegetative stages (mature leaf, seven day old seedling and their roots), 11 reproductive stages (P1–P6 and S1–S5; representing panicle and seed developmental stages, respectively) of rice and three abiotic stress conditions, i.e. cold, salt, and dehydration. Total RNA was isolated from three biological replicates for each stage/treatment and microarray experiments were carried out using 51 Affymetrix Gene Chip Rice Genome Arrays (Gene Expression Omnibus, GEO, platform accession number GPL2025). The raw data (*.cel) files, generated from all the chips were imported to Array Assist 5.5.1 software (Stratagene, USA) for the detailed analysis. Microarray analysis was performed according to Ray et al., 2007 [65]. The microarray expression data have been deposited in the gene expression omnibus (GEO) database at NCBI, under the series accession numbers GSE6893 and GSE6901. For *Arabidopsis*, microarray data was extracted from Genevestigator (http://www.genevestigator.ethz.ch/at) (ATH:22K chip) for the abiotic stresses (salt, cold and drought) and various vegetative and reproductive stage tissues.

### Quantitative expression analysis by real time PCR

Real time PCR was done to validate the microarray data for a few selected genes, showing significant differential expression pattern under abiotic stress conditions. The primers were design for all the selected genes preferentially, from 3′ end, using PRIMER EXPRESS (PE Applied Biosystems, USA), with default parameters. Primers were checked using BLAST tool of NCBI and dissociation curve analysis after the PCR reaction for their specificity (Table S4). First strand cDNA was prepared from 4 µg of DNase treated total RNA, in 100 µl of reaction volume using high-capacity cDNA Archive kit (Applied Biosystems, USA). KAPA SYBR FAST Master Mix (KAPABIOSYSTEMS, USA) was used to determine the expression levels for the genes in ABI Prism 7000 Sequence detection System (Applied Biosystems, USA). Biological duplicates of each sample were taken for the analysis. The average Ct values were calculated by taking the average of three technical replicates for each sample. The cDNA variance among samples was normalized using *ACTIN*, as the endogenous control. Relative expression values were calculated by ΔΔCt method and normalized the data against the maximum average expression value from microarray.

### Preparation of GFP fusion constructs for sub-cellular localization

ORF (lacking stop codon) of respective PLAs were amplified from stress treated cDNA of rice (IR64) with gene specific primers (Table S5), using iProof high-fidelity DNA polymerase (Bio-Rad). GFP-OssPLA_2_α construct was prepared by cloning the amplified *OssPLA_2_α* CDS in Gateway® entry vector pENTR-D/TOPO (Invitrogen) and subsequent mobilization to gateway compatible binary vector pSITE 2CA [66], by Gateway cloning protocol (Invitrogen). Whereas, to prepare OspPLA-IIIδ-GFP and OsPLA_1_-IIβ-GFP construct, CDS of *OspPLA-IIIδ* and *OsPLA_1_-IIβ* was cloned between *Bam*HI and *Sal*I restriction sites of binary vector, pGPTVII.GFP.Kan [67]. Successful preparation of all the constructs was confirmed by sequencing. The 35S CaMV promoter regulates the expression of cloned gene in both the vectors.

### Particle bombardment and confocal microscopy

Epidermal peels were taken from the surface of the spring onions bulb leaves and placed on 1/2 MS-agar plates, supplemented with 1% sucrose. For localization, approximately 2.5 µg of construct plasmid, and for co-localization 2.5 µg of both organelle marker [38] and PLA construct plasmids were coated onto 1 µm gold particle (Bio-Rad) and introduced transiently into onion epidermal cells by microprojectile bombardment, using a Bio-Rad PDS/1000 helium-driven particle accelerator, as per manufacturer's instructions. All the samples were incubated for 16–24 h under dark conditions at 28°C before microscopic analysis. Transiently transformed epidermal peels were analyzed in TCS SP5 laser scanning confocal microscope (Leica, Germany) for fluorescence detection. GFP signals were detected between 505–550 nm after exciting with 488 nm laser, while RFP signals detected at 600–630 nm laser band width range with excitation at 543 nm. For the co-localization experiments, sequential scanning was done for both the channels and then merged together to shows overlapping signals. All the images were further processed using Leica LAS AF Lite software.

## Supporting Information

Figure S1 **Expression profile of** ***Arabidopsis*** **PLA superfamily under abiotic stresses.** Expression heatmap, extracted from Genevestigator database for PLA_1_, pPLA and sPLA_2_ classes indicating the transcript level under salt, drought and cold stresses. The color scale at the bottom of heat map is given in log_2_ scale indicating the fold up-or down-regulation w.r.t. control samples.
(TIF)

Figure S2 **Expression profile of** ***Arabidopsis*** **PLA superfamily in various tissues.** Expression heatmap for PLA_1_, pPLA and sPLA_2_ classes indicating the transcript level of genes in various tissues such as seedling, leaf, root (vegetative) and seed and floral organs (reproductive). Reference scale is given at the bottom of the heatmap.
(TIF)

Table S1 **OsPLAs in segmental and tandem duplication.**
(XLSX)

Table S2 **Microarray expression data for OsPLAs under three abiotic stresses.**
(XLSX)

Table S3 **Microarray expression data for OsPLAs during development.**
(XLSX)

Table S4 **List of primers used for real time PCR expression analysis of OsPLAs.**
(XLSX)

Table S5 **List of primers used for cloning of OsPLAs for subcellular localization.**
(XLSX)
