# Supplementary figures and images for: Rice Phospholipase A Superfamily: Organization, Phylogenetic and Expression Analysis during Abiotic Stresses and Development
Source: PLoS One. 2012 Feb 17;7(2):e30947. doi: 10.1371/journal.pone.0030947 (PMC3281901; doi:10.1371/journal.pone.0030947)

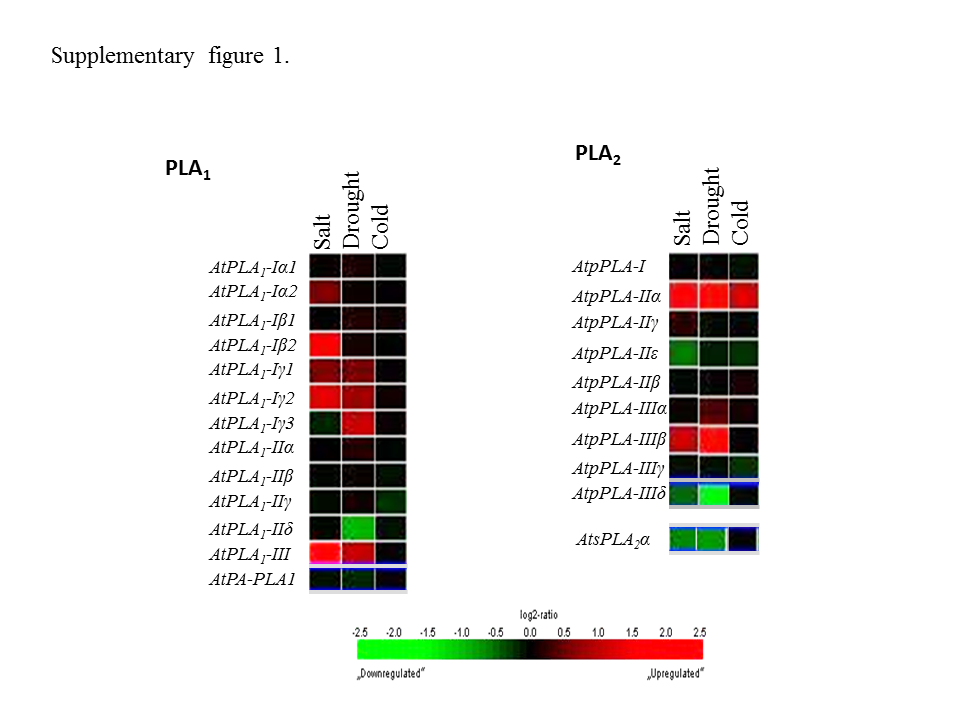

Supplement: Figure S1 — Expression profile of Arabidopsis PLA superfamily under abiotic stresses. Expression heatmap, extracted from Genevestigator database for PLA1, pPLA and sPLA2 classes indicating the transcript level under salt, drought and cold stresses. The color scale at the bottom of heat map is given in log2 scale indicating the fold up-or down-regulation w.r.t. control samples. (TIF) [file pone.0030947.s001.tif]

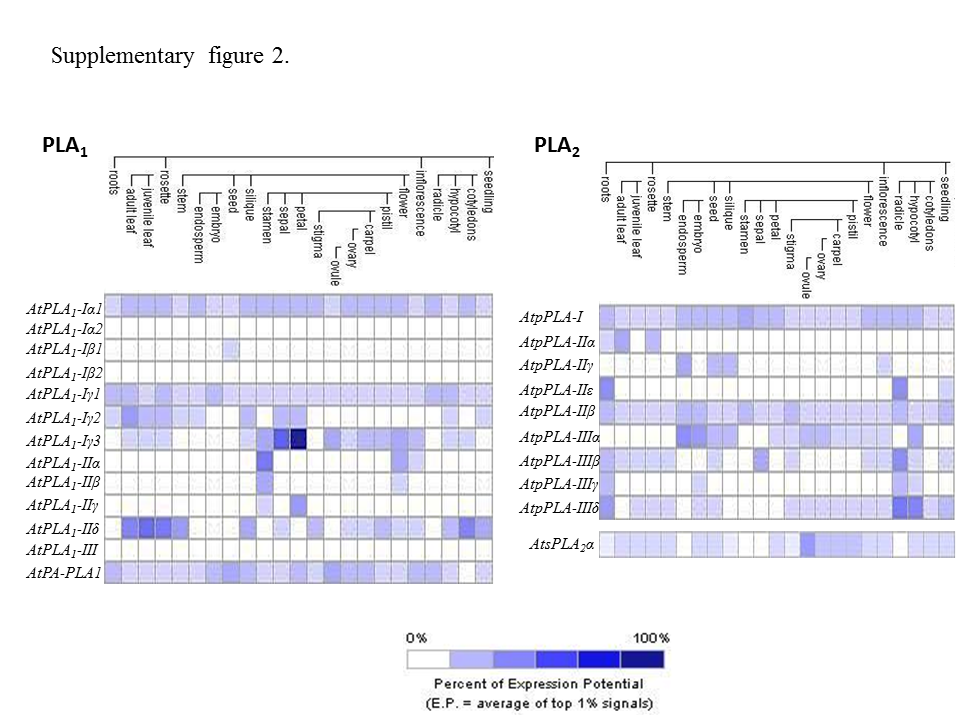

Supplement: Figure S2 — Expression profile of Arabidopsis PLA superfamily in various tissues. Expression heatmap for PLA1, pPLA and sPLA2 classes indicating the transcript level of genes in various tissues such as seedling, leaf, root (vegetative) and seed and floral organs (reproductive). Reference scale is given at the bottom of the heatmap. (TIF) [file pone.0030947.s002.tif]
